# MEMS-Based Micro Sensors for Measuring the Tiny Forces Acting on Insects

**DOI:** 10.3390/s22208018

**Published:** 2022-10-20

**Authors:** Hidetoshi Takahashi

**Affiliations:** Department of Mechanical Engineering, Faculty of Science and Technology, Keio University, 3-14-1 Hiyoshi, Kouhoku-ku, Yokohama 223-8522, Japan; htakahashi@mech.keio.ac.jp; Tel.: +81-4-5566-1847

**Keywords:** insect, MEMS, force plate, ground reaction force, probe sensor, flight force

## Abstract

Small insects perform agile locomotion, such as running, jumping, and flying. Recently, many robots, inspired by such insect performance, have been developed and are expected to be smaller and more maneuverable than conventional robots. For the development of insect-inspired robots, understanding the mechanical dynamics of the target insect is important. However, evaluating the dynamics via conventional commercialized force sensors is difficult because the exerted force and insect itself are tiny in strength and size. Here, we review force sensor devices, especially fabricated for measuring the tiny forces acting on insects during locomotion. As the force sensor, micro-force plates for measuring the ground reaction force and micro-force probes for measuring the flying force have mainly been developed. In addition, many such sensors have been fabricated via a microelectromechanical system (MEMS) process, due to the process precision and high sensitivity. In this review, we focus on the sensing principle, design guide, fabrication process, and measurement method of each sensor, as well as the technical challenges in each method. Finally, the common process flow of the development of specialized MEMS sensors is briefly discussed.

## 1. Introduction

In recent years, many robots, inspired by small animals, including insects with agile locomotion, have been studied [1,2,3,4,5,6]. Examples of microrobots include micro-air vehicles (MAVs) with flapping wings [7,8], walking and jumping robots [9,10,11], and swimming robots [12,13], based on insects and microorganisms. These bioinspired robots are smaller and more maneuverable than conventional robots, so they are expected to operate effectively, even in areas where humans cannot enter. As the common issue, when taking inspiration from small animals, such as insects, how or what kinds of features to take inspiration from is important. One of the most fundamental issues is to duplicate the shape or the movement of the target small insects, which allows us to reproduce their locomotion, to some extent, by a robot. However, to systematically establish an insect-inspired method, understanding not only the superficial shapes and movements, but also the mechanical dynamics, is important. One of the reasons is that the dominant forces that form locomotion differ, according to body shape and size [14], and the conventional mechanical system cannot be intuitively applied.

Provided that we focus on the specific locomotion and corresponding forces of small insects, clarifying, for example, on the aerodynamic force acting on flapping wings, in the case of flapping flight, is important. In the case of swimming, the force acting on a body is similar to that in the flapping flight case, and only the fluid is different. In the case of walking, the ground reaction force (GRF) and ground adhesion force (GAF) are important. However, directly measuring such forces has been difficult because these forces are tiny, and the target insects themselves are small. For example, the aerodynamic force per area of flapping wings of a butterfly is several Pa, and the GRF of ants is several tens of μN; we can easily approximate these values from their masses and body shapes. In addition, such forces sometimes change rapidly; for example, the flapping frequency of a fruit fly is approximately 200 Hz. Thus, the indirect methods of estimating such forces have been widely studied. The most basic measurement method is to calculate the acceleration from video images and convert it into the force. Additionally, in the case of flapping flight, the main methods of force estimation are computational fluid dynamics (CFD) [15,16,17] and the use of robotic wings, with the Reynolds numbers matching those of actual insects [18,19,20].

Alternatively, measurement science and technology, utilizing, for example, microelectromechanical systems (MEMS), has been developed, and we can easily obtain a tool to measure tiny forces [21,22,23]. Accordingly, using MEMS-based force sensors, recent studies have reported direct methods for measuring the force exerted by small animals, such as insects, which was difficult in previous studies [24,25,26,27,28,29,30,31,32,33,34,35,36,37]. In these studies, the force sensor itself has been specially developed for the target insect, including the sensor size and structure, force range and resolution, time resolution, resonant frequency, and so on.

Here, this paper presents a review of the research on these measurement systems, including critical comments. First, we briefly introduce the development of MEMS sensors. Then, as one of the most widely developed sensors, micro-force plates for measuring the GRF and GAF are introduced. Next, we show the methods for measuring the aerodynamic force of flapping flight, including the total aerodynamic force acting on an insect body and the differential pressure between wing surfaces. These two forces are the major forces that have been measured in many studies, but other forces are also shown in brief. This review focuses on MEMS sensors to measure the forces exerted by small insects and includes a wide range of compliant small animals, cells, and sensor structures because these studies are not completely separated, but are successional to each other and useful for understanding the methodology to measure the tiny forces that act on insects with MEMS-based micro technologies.

## 2. Development of MEMS Technology

MEMS technology is one of the most developed research areas in the field of mechanical and electronic engineering in recent years. MEMS is defined as a whole range of microdevices that integrate various functions, such as mechanical, electronic, optical, and chemical functions. MEMS devices are fabricated on wafers with layered materials, such as metals; their size is generally on the order of mm or less in total length, and their components are usually on the order of μm. The limitation of the component size is mainly due to the wavelength of ultraviolet (UV) light in photolithography, which is one of the typical processes used to transfer a geometric pattern from a photomask to a photoresist on the wafer.

MEMS processes, other than photolithography, include, for example, deposition processes and etching processes. Due to the photolithography and other process characteristics, a number of chips of the same MEMS device can be fabricated on the same wafer in one process. For example, a 1 mm square chip can be fabricated in units of several thousand on a 6-inch wafer. As the processing wafer, a Si wafer or a silicon on insulator (SOI) wafer are common materials for the MEMS process. Industrially, the MEMS process is manufactured with 4-, 6-, or 8-inch wafers.

Among MEMS devices, force sensors are one of the typical devices. One of the earliest MEMS force sensors was, for example, a semiconductor pressure sensor [38], which consisted of piezoresistors formed on a Si membrane as a sensing element. Since then, accelerometers/gyroscopes [39,40,41], etc., have been developed as MEMS physical force sensor devices. Most of these sensors consist of specially designed Si structure and sensing elements. As the sensing elements, the piezoresistive, piezoelectric, and capacitive elements are mainly used [42,43,44]. For example, capacitive accelerometers are mainly composed of proof mass and comb structures. In the case of industrial high-performance MEMS sensors, the sensing circuit, including an amplifier, is sometimes additionally integrated on the same sensor chip. In contrast, in the case of one-of-a-kind custom sensor devices, such as for insect measurement, the circuit is separate and configured outside the MEMS sensor chip. Then, the circuit must be placed close to the sensor chip to reduce the electrical noise level. For example, in the case of a piezoresistive sensor, the piezoresistive element is incorporated into a Wheatstone bridge circuit, which converts the resistance change into a voltage change. Then, the voltage change is amplified via an instrumentation amplifier. Because the force exerted by tiny insects is minute, the sensor signal is also weak. In general, the fractional resistance change, as small as the order of 10^−5^, is a detectable threshold. Thus, when designing the force resolution, the fractional resistance change of that magnitude should be the minimum measurement force.

In recent years, flexible and stretchable MEMS sensors, not based on Si, have also been developed [45,46]. In these devices, polydimethylsiloxane (PDMS) and hydrogels are widely used as device materials, and many studies have been reported in the microfluidics and bioMEMS fields. To form the device structure with these materials, especially PDMS, a moulding process (moulding models are fabricated by a common MEMS process) is usually conducted [47,48]. Hydrogels can be adapted for photolithography by mixing with UV-curing materials. Similar to other fabrication methods, by using microflow channels to mix several liquid materials, the material is chemically cured into the desired shape [49].

To understand the MEMS sensor for the force measurement of insects, here, we briefly describe the common fabrication process. First, the sensor structure, including the position of the sensing element or the metal wiring, is designed according to the required specifications. Simulation or CAD software is used for the design. At the same time, the starting material is determined. If the starting material is an SOI wafer, then the thickness of the device Si layer usually becomes the device thickness. The thickness of the device Si layer is fixed, to some extent, commercially; thus, we need to design the sensor structure according to an available wafer. After determining the design, the photomasks are prepared. The number of photomasks is determined by the number of patterning layers. Additionally, preprocessing of the SOI wafer is performed, such as forming a piezoresistive layer with ion implantation and depositing a metal layer with sputtering equipment. Then, the photomask pattern is transferred to the photoresist coated on the wafer by photolithography. Each layer is etched according to the photoresist pattern. Normally, the metal layers are etched via a wet etching process, while the Si layers are etched via a dry etching process with an inductively coupled plasma (ICP)-reactive ion etching (RIE). After fabrication is conducted, the device chip is picked from the wafer via a wafer dicing process. The device chip is attached to a substrate and wire bonded to a pad for electrical connection. A MEMS device is fabricated through a series of these processes.

## 3. Measurement of the GRF/GAF

### 3.1. Introduction to Force Plates

One of the most common forces that animals exert during locomotion is the force acting at the boundary surface between legs and the ground. If the force direction acts as repulsion, then this force is the GRF, and if it acts as attraction, then this force is the GAF. Since humans also receive a GRF from the ground during walking and running, the GRF has been studied for a long time in the biomechanics field [50,51,52]. In these studies, force plates have been used to quantitatively measure the GRF. A force plate is similar to a scale, in principle; however, it measures the dynamic force, while a scale measures the static force, “body weight”. Currently, the general force plate consists of a square plate and force sensor parts that support the plate on four corners of the plate. Each force sensor part is capable of measuring the 6-axis force (triaxial force and triaxial torque), so that the GRF can be detected three-dimensionally when a single sole lands on the plate surface. At this moment, the centre of the force can also be calculated based on the output of the four force sensor parts. By placing force plates on a road surface, such that each sole lands on the corresponding plate at the right step, we can measure the GRF changes during walking and running.

Force plates for humans are designed and manufactured to adjust to human gait, sole size, and weight. To match the human legs, the plate size is approximately 50 cm square. The measurable force range is designed to be, for example, approximately 3000 N. Force plates for humans are adaptable to similar sized animals, such as dogs [53,54]. However, for small animals, such as insects, such force plates are not suitable. Developing a force plate specialized to the target small animal, so that the GRF can be measured during locomotion, is necessary.

### 3.2. Early Force Plates for Insects

The development of force plates for nonhuman animals has been proceeding since the early 1970s–1980s [55,56,57,58,59]. As a force plate for small animals, for example, N. C. Heglund developed an inexpensive force plate that can measure GRFs in the biaxial directions [58]. The author noted that the force plate should satisfy some requirements, in addition to the fundamental specifications: (i) low “crosstalk” between the force components; (ii) a response independence, regarding where on the plate surface the force is exerted; (iii) a resonant frequency sufficiently higher than the frequency of animal locomotion; and (iv) a sufficient safety margin to protect the plate and the target animal. Although the force plate developed in the literature was not fabricated with the MEMS process, the design guidelines can be applied to many other force plates, including MEMS force plates. In the literature, force plates of three sizes were developed. As the smallest force plate, a 25 cm square and 300 g plate, with a honeycomb structure inside, was supported by four beams with horizontal and vertical notches. Strain gauges were attached to each notched part, for a total of eight strain gauges. The force, applied to the plate in two directions, was measured from the fractional resistance changes of these strain gauges. The measurement range, resolution, and resonant frequency were 26.5 N, 0.1 N, and 170 Hz, respectively (Table 1). The forward horizontal and vertical GRFs exerted by a 112 g kangaroo rat (*Dipodomys spectabolis*) were demonstrated to be measured for one step when the rat hopped on the plate on both feet. These structures were not yet suitable for measuring the GRF of insects in the g-order or mg-order in the measurable range and force resolution.

**Figure 1:**
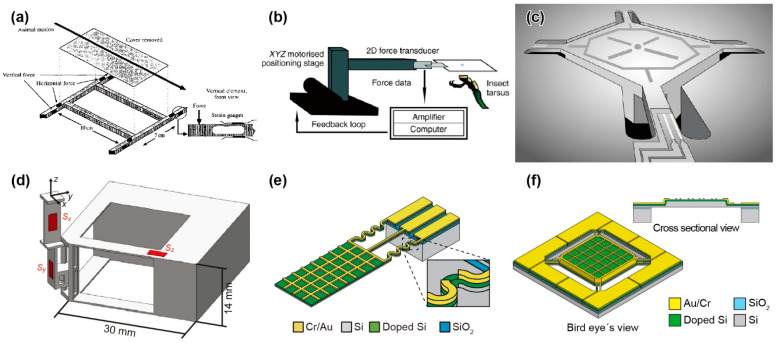
Schematic images of micro-force plates for insects. (**a**) Biaxial force plate for a cockroach. Reprinted/adapted with permission from Ref. [62]. 2022, Company of Biologists Ltd. (**b**) Biaxial force plate for measuring the GAF of soles. Reprinted/adapted with permission from Ref. [69]. 2022, Company of Biologists Ltd. (**c**) Triaxial MEMS force plate for a cockroach. Reprinted/adapted with permission from Ref. [24]. 2022, IEEE. (**d**) Triaxial 3D-printed force plate for an ant. Reprinted/adapted with permission from [76]. 2022, Company of Biologists Ltd. (**e**) Biaxial MEMS force plate array for an ant [25]. (**f**) Uniaxial MEMS force plate for a fruit fly [26].

Since the 1990s, cockroaches and geckos have been chosen as target small animals for gait analysis. Cockroaches run at sufficiently high speeds using wave gait and tripod gait [60]. In addition, they can move through vertical walls and along flat ground, with similar agile performance, by using the GRF and GAF with the corresponding legs. Geckos also generate a GAF by the van der Waals force of the microhair structure on their soles to walk on both the ground and vertical walls [61]. R. J. Full et al. developed a force plate for a cockroach, based on the above design guidelines [62,63,64]. The force plate was composed of brass spring blades and a 10.7 cm × 7 cm wooden cover, as shown in Figure 1a. Semiconductor strain gauges were mounted vertically and horizontally on the spring blade corners to measure the corresponding vertical and horizontal deformation of the beams caused by the forces exerted when a cockroach moved over the cover. The crosstalk between the vertical and horizontal GRFs was less than 2%. Temperature compensation was performed using the two-gauge method at each corner. The measurement range, resolution, and resonant frequency were 0.1 N, 0.001 N, and 400–650 Hz, respectively (Table 1).

**Figure 2:**
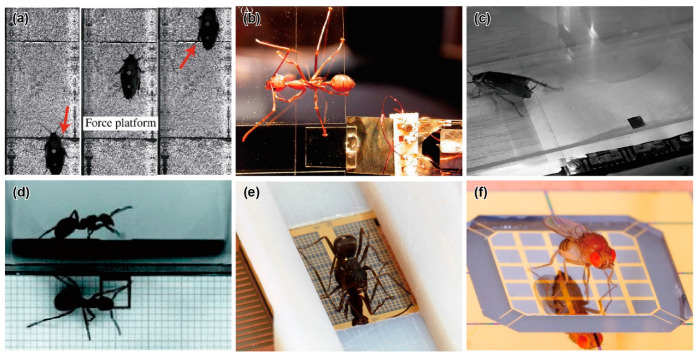
Photographs of insects located on specially fabricated force plates. (**a**) Climbing cockroach on a force plate. Reprinted/adapted with permission from [65]. 2022, Company of Biologists Ltd. (**b**) Weaver ant on a vertical transparent force plate [72]. (**c**) Running cockroach on a MEMS force plate. Reprinted/adapted with permission from [24]. 2022, IEEE. (**d**) Wood ant on a 3D-printed force plate. Reprinted/adapted with permission from [76]. 2022, Company of Biologists Ltd. (**e**) Running ant on a MEMS force plate array [25]. (**f**) Jumping fruit fly on a MEMS force plate [26].

The authors measured the GRFs of a running cockroach (*Blaberus discoidalis*), with a mass and a body length of approximately 1~2 g and 5 cm, respectively. The force plate was sufficiently large, so that the GRF was measured not only when a single leg was positioned, but also when all grounded legs were positioned on the plate. Thus, the total GRF for several steps could be measured. The experimental results showed that the vertical total GRF oscillated around the body weight, according to the gait steps of the running cockroach. The authors’ group also demonstrated that the force plate could be modified to measure the GRFs (including GAFs) of cockroaches (*Blaberus discoidalis*) of 2.9 g and geckos (*Hemidactylus garnotii*) of 1.9 g climbing up a vertical wall, as shown in Figure 2a [65,66]. The developed force plates were primitive; thus, the sensitivities were on the order of mN. However, these became a design guideline for subsequent micro-force plates; for example, the plate frame and supporting beams were united. Recently, another research group has used a similar force plate for climbing cockroaches [67]. The plate surface was composed of a plastic material with holes, which enables cockroaches to engage their claws and use their adhesive pads for climbing.

These force plates were designed to measure the total GRF and are not suitable for the GRF of each individual leg. Since geckos have four legs, there is a chance to measure the single GRF of their front legs when landing on the plate and their hind legs when landing off. Meanwhile, it cannot measure the middle legs of a six-legged insect.

### 3.3. Force Plates for Tiny GAFs

Micro-force plates are also utilized to measure the “static” GAF of a single sole of insects. By pulling the insect’s body, when the sole adheres to the plate, the maximum GAF is measured as the force at the moment the sole separates. Researchers have focused on not only the static GAF that a single sole structure can produce, but also the dynamically changing GAF during the climbing motion. The micro-force plates developed for GAFs also have many characteristics in common with other force plates.

The W. Federle group developed a custom hand force plate to investigate the single leg GAFs of small insects, such as cockroaches and ants [68,69,70,71,72]. The force plate was composed of a glass plate and a supporting 2D bending beam, which measured two directional forces (friction and adhesion forces) by strain gauges arranged orthogonally [68,69], as shown in Figure 1b. The method of arranging biaxial strain gauges is common to the plates described above, but the force plate was defined as a cantilever structure, unlike the plate with all edges fixed. In the case of a cantilever structure and a simple gauge, the response may vary, depending on the applied force position, even if the force is the same. However, the leg position is thought to be roughly controllable, or a correction factor based on the position can be used. The size of the glass plate was 5 mm square, and the highest sensitivity for both directional forces was 5.6 μN (Table 1). Recently, they have developed a force plate with a 2D fibre-optic transducer as the sensing element [73]. Thus, the independence of the two axes was improved by using optical measurement. The spring constant can also be easily designed by separating the mechanical element and sensing element.

Additionally, one of the features of the force plate was that the glass plate was transparent, so the sole adhesive surface could be observed from the backside via a microscope. Furthermore, the measurement could be conducted with force feedback control by fixing the force plate to an *xyz* stage. In addition, the W. Federle group utilized a MEMS structure to realize the rough surface of a force plate [74]. An SU-8 (thick negative photoresist) micropillar array was fabricated as the moulding master via a photolithograph process, and an epoxy pillar structure was arrayed on a glass plate via a PDMS moulding process. Then, a force plate with an “organized” rough surface was realized. Due to the MEMS photolithograph process, identically shaped microstructures could easily be arrayed on a surface.

One of the small insects that the W. Federle group measured was a weaver ant (*Oecophylla smaragdina*) of approximately 8 mg [72]. The single leg contact force during climbing was measured via the glass force plate (Figure 2b). The experimental results revealed that the contact force became adhesive and reactive when the corresponding contacting leg was above the centre of mass (CoM) and below the CoM, respectively. In addition, the ants’ legs tended to produce smaller than normal shear forces.

The force plate for static GAF measurement is not required to realize high time responsibility. Therefore, even if such force plate has high sensitivity, its resonant frequency may be low. The measurement also depends on the force applied position, so it is necessary to contact the leg at the same position each time to obtain accurate data. Thus, it may not be suitable for dynamic GAF or GRF measurement during motions, such as running, of which we cannot control the landing positions. In addition, the force plate is mainly made of transparent glass to observe the sole from the backside. However, glass is not suitable for microfabrication, including the MEMS process; thus, it must be handmade. If both transparency and microfabrication are required, a MEMS-based Si plate with infrared light or fabrication with transparent film material, such as SU-8, is considered.

**Table 1:** Specifications of the developed force plates. The force plates were designed according to the size and motion of the measurement target.

	Target Animal/Insect	Plate Size	Sensing Element	Force Direction	Force Resolution	Resonant Frequency
N. C. Heglund (1981) [58]	Kangaroo rat 112 g	25 × 25 cm	Strain gauge	*x*, *z*	0.1 N	170 Hz
R. J. Full et al. (1990, 1991, 1991) [62,63,64]	Cockroach 2.3 g	10.7 × 7 cm	Semiconductor strain gauge	*x*, *z*	0.001 N	400–650 Hz
T. Endlein and W. Federle (2015) [72]	Ant 8.2 mg	5 mm square	Semiconductor strain gauge	*x*, *z*	5.6 μN	80 Hz
M. S. Bartsch et al. (2007) [24]	Cockroach 0.47 g	5.3 mm square	Piezoresistive element	*x*, *y*, *z*	2.2 nN/Hz^1/2^ at 1 kHz	0.9–9 kHz
S. Kohyama et al. (2018) [29]	Ant 8 mg	15 × 7.5 mm	Piezoresistive element	*z*	0.5 μN	No data
L. Reinhardt et al. (2009, 2014) [75,76]	Ant ~20 mg	4 mm square	Semiconductor strain gauge	*x*, *y*, *z*	10 μN	200 Hz
H. Takahashi et al. (2014) [25]	Ant 3 mg	2 × 1 mm array	Piezoresistive element	*x*, *z*	1 μN	560 Hz
H. Takahashi et al. (2017) [28]	Fruit fly 1.0 mg	5 mm square	Piezoresistive element	*z*	1 μN	1.2 kHz

### 3.4. MEMS Force Plates for a Cockroach and an Ant

M. S. Bartsch et al. reported a MEMS-based force plate, which was a first-generation multi-axis MEMS force sensor [24]. The force plate was designed to measure the single leg three-axis GRF of a *Blaberus* cockroach. The force plate consisted of a 5.3 mm square plate supported by four beams (555 μm long and 70 μm wide), as shown in Figure 1c. Piezoresistive elements were formed on the surface of each beam to measure both the vertical and horizontal force components. Then, the fractional resistance changes of the four piezoresistive elements could be used to determine the three-axis forces exerted on the plate surface. The fabrication process of the force plate began with an SOI wafer with a 15–75 μm thick device Si layer. After the piezoresistive layer and wiring layer formation processes, the force plate structure was formed by etching both the device Si and handle Si layers by deep RIE (DRIE) from the top and bottom, respectively. All of the handle Si layer at the beam sections was etched, so that the beams were relatively deformable. There were also etching holes in the handle Si layer on the plate backside to reduce its mass and to maintain a sufficiently high resonant frequency. The fabricated force plate was calibrated to both vertical and horizontal forces by connecting the piezoresistive elements to a bridge and amplifier circuit. The force plate, with an 18 μm thick device Si layer, realized sensitivities of 55 and 12 V/N for vertical and horizontal forces, respectively, and the vertical force resolution reached 2.2 nN/Hz^1/2^ at 1 kHz (Table 1). The authors demonstrated the measurement of the single leg GRF of a cockroach (*Periplaneta americana*) of 0.47 g, as shown in Figure 2c. The demonstration showed that its vertical GRF, which was in the gravity direction, reached 30% of its weight. This value was reasonable, assuming that the cockroach used a tripod gait. In contrast to the vertical GRF, the maximum horizontal GRFs were approximately 5~10% of its weight. The authors concluded that the developed force plate realized significantly higher performance than those previously available, due to MEMS technology. The force plate was designed to measure in triaxial GRF. However, the piezoresistive layer, which was the sensing element, was formed on only the surface Si layer, due to the limitation of the conventional MEMS process. Thus, the beam supporting the plate must be significantly thin to detect in-plane force *F*_x_ and *F*_y_. Thin beams are fragile and require the most careful attention in fabrication and experimentation. The sensor design was applicable to measure the GRF of “g” mass cockroaches. However, it is considered difficult to measure “mg” mass insects by this design.

The H. Takahashi and I. Shimoyama group developed a MEMS force plate for a walking ant [29]. They focused on the z-axis vertical GRF during running and, thus, designed a MEMS force plate that could detect only the z-axis force with high sensitivity. A MEMS piezoresistive cantilever structure, which was fabricated separately from the plate, was used for the sensing element. The cantilever was also fabricated on an SOI wafer, with a similar process to that for the above force plate. The four fabricated cantilever structures were fixed on a substrate. Then, a 15 mm × 7.5 mm glass plate was mounted on the top, with glue to support the four corners with the tips of the four cantilevers. The thickness of the cantilever was designed to be 5 μm for a high force resolution of less than 1 μN (Table 1). The fabricated force plate was embedded in an experimental runway of ants. Then, the total GRF was measured when an ant (*Messor aciculatus*) of 8.2 mg ran across on the plate. The measured total GRF showed little oscillation, unlike the experimental results for cockroaches [62]. The results indicated that a different force mechanism worked in “mg” mass ants than in “g” mass cockroaches. The sensing element was fabricated in the MEMS process; however, the assembling process was manual. The reason for this process is that the sensing element was significantly thin, to realize the high sensitivity. It is quite difficult to fabricate a large plate and a sensitive beam in one single process, such as the force plate of the cockroach [24]. Additionally, it is difficult, in principle, to expand to multi-axis force plates, due to the aspect ratio of the supporting cantilevers.

### 3.5. D-Printed Force Plates for an Ant

Similar to MEMS technology, 3D printer technology, which can fabricate a micro-3D structure, has been rapidly developed in recent years. These 3D printers are suitable for producing custom-made structures; thus, they are applicable to force plates for small insects. The L. Reinhardt group developed a force plate for ants, the frame of which was fabricated by a 3D printer [75,76,77,78]. The frame was a cantilever structure with mutually orthogonal surfaces, similar to the force plate, as shown in Figure 1d. A 4 mm square plate was placed at the tip of the frame. The frame was built layer-by-layer via a stereolithography-type 3D printer using a polycarbonate-like material. The thinnest beam in the frame had a thickness of 0.4 mm. A semiconductor strain gauge was attached in each direction in the largest strain area of the frame, so that the three-dimensional force was measured with low crosstalk. The force resolution and resonant frequency reached 10 μN and 200 Hz, respectively (Table 1). Then, the calibrated force plate was embedded in the same runway as above. The GRF was measured when a wood ant (*Formica polyctena*) of approximately 20 mg ran on the runway and a single leg touched the plate surface by chance (Figure 2d). Because the measurement was conducted along three axes, the measured forces in the *x* and *y* directions could be transformed into travelling and lateral forces, according to the ant’s running direction.

As the author noted, the difficulty level of the fabrication process with a 3D printer is low. In addition, the fabricated structure is not easily broken, compared with MEMS Si structures. Meanwhile, 3D printer materials sometimes have lower material stability than MEMS materials, such as Si and precision-machined metals. It is necessary to pay attention to the plastic deformation or Young’s modulus change for long-time use. However, in recent years, 3D printers have been available, with metals and ceramics as materials. Although integrating sensing elements, such as strain gauges, in the printing process is difficult, it has the potential to be utilized by more researchers to produce force plates in the future.

### 3.6. MEMS Force Plate Arrays

**Figure 3:**
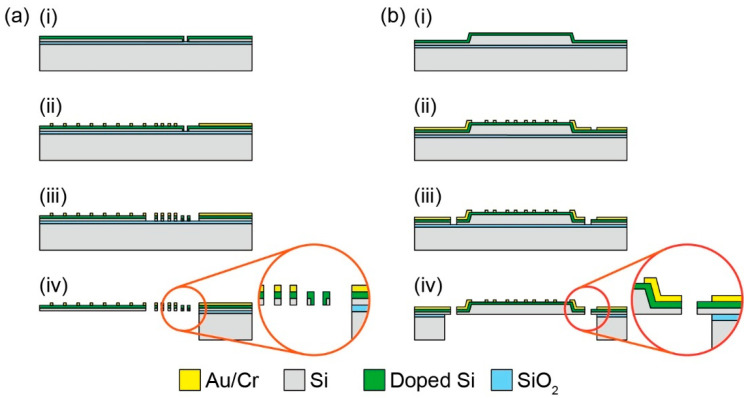
Fabrication process of (**a**) the biaxial MEMS force plate array for an ant [25] and (**b**) uniaxial MEMS force plate for a fruit fly [26]. (**a**-**i**) Doping holes are etched on the device Si layer by DRIE. A piezoresistor layer is formed on both sidewall of the etched holes and device Si surface with rapid thermal diffusion. (**a**-**ii**) Cr/Au layers are patterned on the doped Si layer. (**a**-**iii**) The device Si layer is etched by DRIE. (**a**-**iv**) The handle Si layer is etched by DRIE from the backside. The plate is released by etching the SiO_2_ layer with HF vapor. (**b**-**i**) Mesa structure is formed by wet etching using tetra-methylammonium hydroxide (TMAH) solution. A piezoresistor layer is formed on the device Si surface by rapid thermal diffusion. (**b**-**ii**) Cr/Au layers are deposited and patterned on the doped Si layer. (**b**-**iii**) The device Si layer is etched by DRIE. (**b**-**iv**) The handle Si and SiO_2_ layers are etched by DRIE and HF vapor, respectively.

Although 3D printers can also build micro-force plates for insects, MEMS technology can provide more advanced force plates. The characteristic advantages of MEMS are high sensitivity, due to forming sensing elements in strain concentration areas and high integration of the device structures, which are difficult to manually handle. With these advantages, for example, the H. Takahashi and I. Shimoyama group developed a MEMS force plate array for a running ant [25]. The fabrication process is described in Figure 3a. A Si plate was supported by beams formed with piezoresistive layers on the surface and sidewall, and the overall structure was a cantilever structure, as shown in Figure 1e. The sidewall doping method is not a common MEMS process at present. Compared with the standard surface doping method, sidewall doping is complex. For example, it is necessary to form pre-holes, or the process wafer must be inclined in the ion-implanting equipment. Due to the two-dimensional piezoresistive layer, the *x* and *z* directional forces were measured with low crosstalk. This structure was also applied to the force plate for measuring the cellular traction force [27]. Because of its cantilever structure, it is less fragile than the plates that support four corners, even with the same μN order resolution. However, due to the cantilever structure and gauge, the response varied, depending on the applied force position. This problem was solved by patterning a grid on the plate, calibrating the force on each grid area, and using the calibration values, according to the contacting leg position on the plate. The force resolutions along the two axes were experimentally confirmed to be less than 1 μN. The resonant frequency was 560 Hz (Table 1).

Plates (2 mm × 1 mm) were fabricated on an SOI wafer to be arrayed in parallel, with a narrow gap of 20 μm between each plate. The fabrication process was similar to that described in the previous sections; the difference was that the sidewall doping process was used. The GRFs of three legs on one side of an ant during running could be measured simultaneously by the densely arrayed plates. The array with eight plates was inserted into one side edge of a pathway, where an ant (*Formica japonica*), whose body length and weight were 5 mm and 2.7 mg, respectively, ran. The maximum *z*-axis GRF of each leg was approximately 10 μN, which was equivalent to one-third of the body weight. While each GRF varied, according to the time, the total value was similar to half the body weight. The *x*-axis GRFs of the front and hind legs were negative and positive, respectively. The GRF of the middle leg changed from a negative to a positive value. At this moment, the total value was close to zero. The measured GRF was thought to be one of the characteristics of an ant, including the low vibration of the total GRF, which was indicated by one large force plate [29].

In the literature, the array was inserted only into one side, but the measurement can be conducted for both sides, in principle, as shown in Figure 2e. The force plate array encompasses both the one that measures the total GRF and the one that measures a single leg’s GRF; i.e., the simultaneous GRF distribution is obtained from the measurement data. However, if more than one leg is positioned on a single plate, then the GRF measurement cannot be conducted. Thus, designing a plate array according to the gait of a target insect is desirable. Although the fabricated force plate was a two-axis sensor, it can be expanded to measure three axes by increasing the number of sensing beams, in principle.

### 3.7. High Force and Time Resolution MEMS Force Plates

The jumping, including taking off and landing, of flying insects, is one of the significant types of locomotion in which the GRF changes dramatically. In particular, there is the tendency that the smaller the body size is, the quicker the jumping motion becomes. A force plate for measuring the GRF of such a small insect’s jumping motion is required to realize a high force resolution less than the body weight and a high resonant frequency above the jumping motion frequency.

The H. Takahashi and I. Shimoyama group developed a MEMS force plate for a taking-off and landing fruit fly [26,28]. A 5 mm square plate was supported by four beams, as shown in Figure 1f. The fabrication process is described in Figure 3b. Piezoresistive elements were formed on the surface of each beam to measure the vertical GRF. The sensor structure was similar to the force plate for a cockroach, as described in Section 3.2, but there was a large difference in the thickness; the plate and supporting beams of the force plate for the taking-off motion were 20 μm and 10 μm, respectively. The mesa structure formed by the differential thickness resulted in an accurate force measurement, regardless of the point where the force was applied. To realize such a thickness, the force plate was fabricated with a mesa structure on a device Si layer, which means that a handle Si layer was not used for the plate. The fabricated force plate realized both a force resolution less than 1 μN and a resonant frequency higher than 1 kHz (Table 1). The calibrated force plate was attached to a chamber, and fruit flies (*Drosophila melanogaster*) were placed in the chamber. The GRF was measured when a fruit fly voluntarily took off from the plate surface (Figure 2f). The fruit flies exerted a GRF up to 10 times larger than their body weight during the taking-off motion. The GRF was also measured when a fruit fly voluntarily landed on the plate.

The developed force plate was specialized for an extremely small insect, the fruit fly. To measure the GRF of small insects during their agile motions, the force plate should be sufficiently small, with high performance. MEMS technology is currently considered to be one of the most suitable technologies for developing such devices. Unlike the force plate of the ant of the same group [25], which has a large plate, the plate and supporting beams were fabricated in one process. Thus, the 5-mm-square order is considered the limit of the actual MEMS process to realize both μN order resolution and integrated fabrication. Meanwhile, similar to the ant’s force plate, it is difficult to expand the multi-axial measurement.

### 3.8. Force Plates for Other Animals

We have discussed force plates for tiny GRF and GAF measurement of insects. In a similar manner, dedicated force plates have also been developed for animals other than small insects, such as frogs. Such devices are larger than those for small insects; thus, the utilization of MEMS technology may not be necessary. However, each force plate applies a number of innovative ideas to effectively measure the target. Such ideas are useful for the future development of micro-force plates for insects.

**Figure 4:**
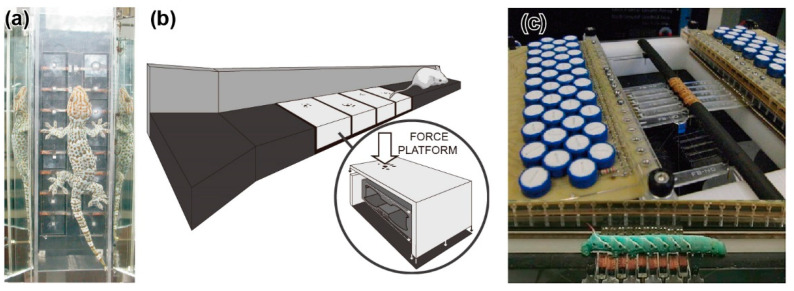
Photographs and schematic image of force plate arrays for a small animal. (**a**) Climbing gecko on a force plate array. Reprinted/adapted with permission from [79]. 2022, Company of Biologists Ltd. (**b**) Force plate array for a running mouse. Reprinted/adapted with permission from [81]. 2022, Elsevier. (**c**) Cantilever-type force plate array for a caterpillar. Reprinted/adapted with permission from [86]. 2022, Elsevier.

As described in Section 3.2, N. C. Heglund developed force plates of three sizes for small animals (kangaroo rats), athletes, and horses. In the 1980s, small triaxial force plates were developed for small animals, for example, cats [59]. Recently, compact force plates that can measure the total GRF of small animals have been commercially available. Force plate arrays have been developed for geckos [79,80], mice [81], frogs [82,83,84], caterpillars [85,86], and so on to measure GRF distributions, as shown in Figure 4.

The force plate array for geckos was composed of 16 force plate elements, with 8 elements on each side of the climbing path [79,80] (Figure 4a). The force plate element was made up of a T-shaped aluminium alloy. A 30 mm square plate was located at the tip of the T-shape. Strain gauges were glued onto three points to measure the triaxial force exerted on the plate. The force resolution was 2 mN and 3 mN, depending on the measurement axis, with a measurable range of 1500 mN. The GRF of a gecko (*Gekko gecko*) of 65.4 g during climbing was demonstrated to be measured with several steps. Similar to the MEMS force plate array for ants [25], the GRF distribution can be obtained. The total GRF was consistent with the GRF measured by the previous type of force plate, consisting of one large plate.

A force plate array for frogs during climbing was similarly developed to measure the GRF. Force plate elements of the same type as those for geckos, described above [79], were densely arranged on a sloped plate [82]. The authors also developed a quasi-cylindrical (octagonal) force plate array resembling a tree [83,84]. A total of 24 force plate elements were arranged in six rows and four columns, with a set of 12 force plates on each leg side. A Chinese flying frog (*Rhacophorus dennysi*) of 156 g was demonstrated to use a clamping grip during climbing motion.

Arranging the force plate elements three-dimensionally, in addition to arraying them on a flat surface, is important not only for small animals, but also for insects. This is because many grounds in nature are not flat for all animals, including insects. For example, ants manoeuvre on twigs as thin as themselves. To implement such shaped “grounds” with a MEMS force plate array, there are several solutions. For example, thin MEMS force plate elements can be arranged on a flexible substrate that can be rounded. Another method is to fabricate sensing elements integrated in a flexible substrate.

### 3.9. Other MEMS Devices

Until now, we have discussed micro-force plates as a method to measure the GRF and GAF of insects. In this section, measurement methods other than force plates are briefly introduced.

#### 3.9.1. MEMS Cantilever

A force plate basically measures the GRF and GAF of a single leg or of all the grounded legs. Even in the case of a single leg, the force generated by its entire sole is measured. Therefore, the sensor shape becomes a “plate” structure, where the entire sole lands. Then, the plate is supported by double-sided beams or cantilever structures. However, a demand arose for measuring more localized forces on the sole. As mentioned in Section 3.2, the microhair structure on the geckos’ soles generates a van der Waals force that helps them walk on both the ground and vertical walls. A force plate can measure the force on the sole, where the microhairs are densely packed, but is unsuitable for measuring the force on a single microhair.

Instead of a force plate, A. Kellar et al. utilized a pointy cantilever-type force sensor to measure the adhesion force of a single gecko microhair [87]. Since a single microhair was the measurement target, the dimensions and force were significantly small. The length and diameter of a single hair are tens of 100 μm and less than 1 μm, respectively. Therefore, a two-axis MEMS force cantilever was used for the measurement [88]. The cantilever consisted of four supporting thin beams and a triangular tip. The length and thickness of the cantilever were approximately 200 μm and 10 μm, respectively. The triangle tip was 1.3 μm in thickness. Due to the sidewall doping method for the supporting beams, similar to the MEMS force plate array for ants [25], the lateral force could be highly sensitively detected. Then, by attaching one microhair of a gecko sole to the triangular tip, the single adhesion force could be measured. The measurement range was several dozens of μN. The direct force measurement revealed the attachment and detachment mechanisms of the single microhair via control of the parallel and perpendicular forces. The W. Federle group also used a similar device to measure the adhesion force of a single microhair of a beetle [89].

Although limited, a cantilever-type force sensor is also applicable for the measurement of the GRF. During measurement, a leg must be positioned on the pointy tip of the cantilever and must not move. A commercially available MEMS cantilever-type force sensor was used to measure the GRF of a fruit fly [90]. The leg of a fixed fruit fly was placed on the cantilever. Then, the pseudo-jumping force was measured by stimulating the brain nerves with electrodes. A similar method was adapted for the measurement of the adhesion force of water spiders, as an in situ dynamic force measurement [91]. The force sensor is composed of a polyvinylidene fluoride (PVDF) cantilever to measure a vertical force. A water spider’s leg was mounted on a fast-moving stage, and the leg tip contacted a water surface, which was placed on the cantilever surface. Then, the adhesion force was measured when the leg was sliding on the water.

As a combination of a plate and a cantilever, K. Matsudaira et al. developed a force sensor device using a MEMS piezoresistive cantilever and a MEMS plate, although the device was designed for force measurement in cells, not insects [92]. Instead of forming the sensing elements on the beam supporting the plate, similar to the MEMS force plate for ants, the force applied to the plate was measured via the cantilever pushing the plate from the side.

In summary, cantilever-type force sensors have the advantage of being highly compatible with the MEMS process, so it is easy to use in research. Meanwhile, similar to the GAF measurements in Section 3.3, there is a restriction that the target leg must be placed to touch the cantilever tip. Therefore, the cantilever type sensor with a thin tip is not suitable for measuring the GRF during dynamic motion.

#### 3.9.2. MEMS Sensor Sheets

The MEMS force plate for insects is based on the force plate for humans, as a structure, i.e., a rigid plate and a supporting structure with sensing elements. Different methods have been developed to measure forces in cells smaller than insects [93]. The most popular methods are micropillar arrays and stretchable substrates with fluorescent beads. In the case of the micropillar array, silicone micropillars, which are smaller than a target cell, are arranged vertically on a transparent substrate. Cells are cultured on the pillars, so that a single cell spans tens to hundreds of pillars. Then, the displacements of each pillar are captured by a microscope and translated to the lateral force exerted on the pillar via simple material mechanics. Because the pillar array is significantly small and dense, the lateral force distribution of a cell can be measured. In the case of the substrate with fluorescent beads, sub-micron fluorescent beads are mixed into silicone or polyacrylamide substrates to serve as markers that can be captured by a microscope. Cells are also cultured on the substrate. Then, the lateral force exerted on the substrate by a cell is calculated by the displacement of the beads. As common points, these methods optically capture the distribution of the local displacement in the cell culture area and convert it from a displacement to a force via material mechanics. This basic measurement principle can be applied not only to cells, but also to small animals and insects.

Using a PDMS rectangular array of micropyramidal bumps, a vertical force sensor sheet was developed for measuring the GRF of a gecko [94]. The PDMS rectangular array was fabricated via a MEMS moulding process from a crystal anisotropic etched Si hole array. The sensor sheet was arranged on a transparent plate, with light incidents from the sides, so that the bumps were on the bottom. When a vertical force was applied to the sheet, the bumps deformed, and the contact area increased. By detecting the increase in the scattered light intensity of the bumps by a camera, the applied force was measured. The bumps were arrayed at 100 μm intervals, which was sufficiently dense for the gecko’s sole size. The measurement performance strongly depended on the camera’s characteristics. For example, the time resolution depended on the camera’s frame rate (which was 60 Hz in the literature.). The measurable range was approximately ±50 kPa. The stress distributions were demonstrated to be not uniform for a climbing gecko (*Gekko gecko*).

Recently, a hydrogel triaxial force sensor sheet was developed for measuring the GRF of much smaller insects, such as mosquitoes [95]. A microgrid pattern of 50 μm pitch was inscribed in the hydrogel sheet. The distortion of the grid was captured by a microscope and transformed into displacement distribution by image analysis. Then, the relationship between the force distribution and the displacement distribution was evaluated by finite element method (FEM) analysis. In the literature, the force resolution was approximately 10 μN in each direction. Triaxial force measurement was demonstrated when a tethered mosquito leg was attached to the sensor sheet by controlling the position via a manipulator. However, the measured values were not yet consistent with those of previous studies, so further investigation was required.

As a similar method, a GRF measurement method based on the shadow method has been reported [96,97]. The sensor system is composed of a transparent elastomer, a light source, a bottom screen, and a CCD camera. When an insect walks on the elastomer surface, the elastomer is distorted by the legs, which leads to a meniscus. Then, the meniscus generates the shadows on the screen, due to the refraction onto the elastomer via a multi-lens system. Since the shadow size is approximately proportional to the meniscus size, the applied force is calculated from the shadow size.

Optical measurement has the advantages of simple fabrication, no wiring or electrode system, and high applicability to distributional measurement. Meanwhile, they are less accurate than electrical sensors, such as piezoresistive sensors. The local deformation of the soft material sheet, such as PDMS, at the point where the leg attaches, is converted into the GRF, but the deformation depends on the contact area and shape. In addition, the three-dimensional forces may be less independent because they interfere with deformation to each other. Therefore, the force calibration of the soft material sheet is quite difficult, while that of the rigid force plate or cantilever is uniquely determined. As a result, the measurement accuracy is low. For the force measurement, microbump structures or microgrids should be formed on soft material sheets to improve the accuracy. A soft material sheet for the GRF measurement of tiny insects was only recently developed, and it should be explored if any geometry will lead to better accuracy in the future. Then, MEMS technology is indispensable for fabricating such devices.

#### 3.9.3. Wearable Force Sensors on the Sole

In recent years, wearable sensors have attracted attention in the field of human biomechanics, and some of these devices measure the GRF during walking and running motion [98,99]. Such devices for measuring the multi-axis GRF are composed of compact multi-axis tactile sensors embedded in the insoles [100] or outsoles [101,102] of shoes. The local GRF distribution, which depends on the number of tactile sensors, can be measured when a target human walks with the shoes on. The advantage of this method is that it does not limit the measurement position, unlike the force plate. If the shoes are worn, then the measurement is available, even in locations where setting up a force plate is difficult, such as on a slope or on ice. Additionally, the tactile sensors must not affect the performance of the insole or outsole; thus, significant smallness and thinness are required. The wearable sensor method was applied not only to a human, but also a horse. Commercially available triaxial force sensors were embedded in a horse shoe [103].

However, such a measurement method can only be applied to animals that wear “shoes”. This is especially unsuitable for geckos, which use the microstructure of the soles, and small insects. A wearable device should not disturb the locomotion; thus, such a device size might be on the sub-micron order, such as a nanoparticle that changes fluorescent light colour with pressure or shear force.

## 4. Measurement of Flight/Aerodynamic Forces

### 4.1. Introduction to Flight and Aerodynamic Forces

The aerodynamic force of the flapping wings of flying animals, which is defined as the force acting at the boundary surface between wings and air, is also one of the most common forces that animals exert during locomotion. Unlike the fixed wing of an airplane, many animals fly by flapping their wings. While some large birds fly with little flapping, small insects constantly flap their wings at a high frequency. For example, the flapping frequencies of a large butterfly and a small fruit fly are 10 Hz and 200 Hz, respectively. The flapping motion produces a pressure difference between the upper and lower surfaces of the wings, which is the aerodynamic force. Here, we define the flight force as the sum of the aerodynamic force applied to the wing and the force due to motion, such as body vibration.

To evaluate these forces quantitatively, experimental measurement via force sensors is an effective solution, as is simulation. Experimental evaluation of the aerodynamic characteristics of airplane wings has been conducted for a long time [104,105]. In the case of airplanes, pressure sensors are attached to the wing surfaces to evaluate the aerodynamic forces. In the case of the flight force, a wing model, sometimes a total airplane model, is attached to the tip of a probe in a wind tunnel. Then, the force applied to the probe is measured with a 6-axis force gauge. The sensors used for these airplane experiments are adjusted to the aerodynamics of very large airplanes with fixed wings.

Similar to the case of the measurement of the flight force of airplane wings, the non-flapping aerodynamic performance of insect, such as a dragonfly, wings has been evaluated by fixation to a sensitive force probe in a wind tunnel [106]. Since the aerodynamic force is in a static state in such a fixed situation, it is relatively easy to measure. However, such sensor systems are still not suitable for dynamically changing forces. The sensor should be specialized, such as pressure sensors to measure the aerodynamic force of insect wings and force probes to measure the flight force during the actual flapping motion.

### 4.2. Early Force Probes for Insects

Here, we introduce force probes for the measurement of the flight force of flapping insects. The measurement principle, using a probe-type sensor, is similar to that for an airplane. An insect is attached to the tip of the probe, so that the flight force acting on the insect body is measured during the flapping motion. Thus, the insect is in a tethered state, which is slightly different from free flight, during the measurement. As a required specification of the force probe, the probe must be sufficiently stiff, so that the resonant frequency is higher than the flapping frequency, even if a target insect is attached to the tip of the probe. In addition, measuring the flapping force in multiple axis directions is desirable because the force direction changes every moment, due to the flapping motion. Of course, the sensitivity should be sufficiently high to measure the tiny flight force, which would be of similar order to the body weight. As the target of experiments with the force probe method, flies have been used for a long time, due to their small size and steady flight motion.

**Figure 5:**
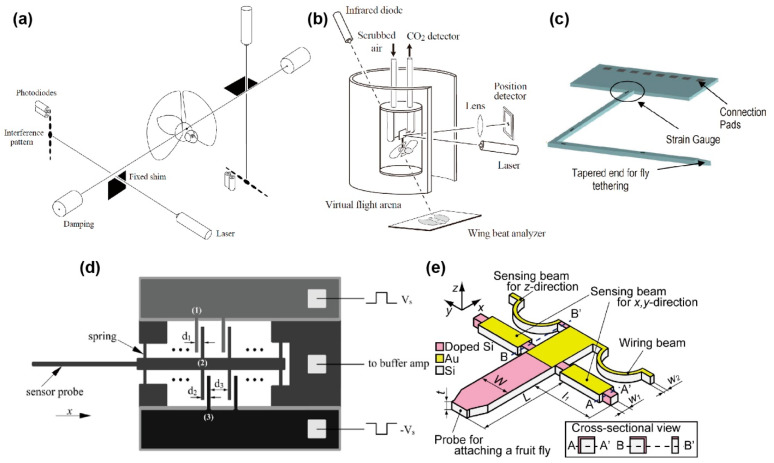
Schematic images of micro-force probes for flies. (**a**) Biaxial force probe using lasers and photodiodes. Reprinted/adapted with permission from [109]. 2022, Company of Biologists Ltd. (**b**) Single force probe using a laser and a photodiode. Reprinted/adapted with permission from [111]. 2022, Company of Biologists Ltd. (**c**) Biaxial MEMS piezoresistive force probe. Reprinted/adapted with permission from [30]. 2022, IEEE. (**d**) Single-axis MEMS capacitive force probe. Reprinted/adapted with permission from [31]. 2022, IEEE. (**e**) Triaxial MEMS piezoresistive force probe. Reprinted/adapted with permission from [37]. 2022, IEEE.

The measurement method with a force probe has been proceeding since the 1980s–1990s [107,108,109]. M. H. Dickinson and K. G. Götz developed biaxial flight force measurement using a tethered wire with lasers and photodiodes, as shown in Figure 5a [109]. A fruit fly (*Drosophila melanogaster*) of approximately 1 mg was tethered to a 60 mm long steel wire, and a shim was adjusted to the boundary of the wire to measure the displacement of the wire along one of the main axes. In the general attachment process, while the fruit fly slept with the application of ice or CO_2_ anaesthesia, the sensor was attached to the dorsal position with UV-curing resin. The laser beam was irradiated into the gap between the wire and shim to generate an interference pattern on the other side. Because the interference pattern changed, according to the gap distance, the displacement of the wire, due to the flight force, was detected based on the light intensity measurement by photodiodes at certain points of the interference pattern. The sensor system realized high stiffness, such that the resonant frequency with a fruit fly was 4.0 kHz (Table 2); however, the wire displacement, due to force, was only 0.29 nm/μN. The authors mentioned that there were several resonances, due to the vibration of the shim or damped oscillation of the tethered fly. Such vibrations were thought to be generated because the wire was exceedingly long, compared to the target displacement. These influences were suppressed by averaging several flapping cycles. The averaged measurement results demonstrated the flight force components were parallel and perpendicular to the longitudinal body axis in one flapping cycle.

M. H. Dickinson et al. also developed a single-axis force probe using a similar principle [110,111]. Instead of using the change in the gap between the wire and the shim, a mirror was attached to the wire. Then, a laser was irradiated on the mirror, and the reflected light was measured by a photodiode (Table 2, Figure 5b). Because the mirror tilted when a force was applied to the wire tip, the reflected light spot shifted. Thus, by measuring the spot displacement, the force could be detected.

The optical method, with lasers and photodiodes, was also used in the force plate in Section 3.3. The optical method has the advantage that the sensing element is constructed without affecting the mechanical property, if the sensor structure is significant large. However, for the sensor structure of a tiny fruit fly, extra shims or mirrors must be installed to the sensor structure, which increases the sensor weight and decreases the resonant frequency. In addition, since the sensor structure is smaller, it is more complicated to build an optical setup. Any difference in the setup will change the response characteristics, so calibration is required each time.

**Figure 6:**
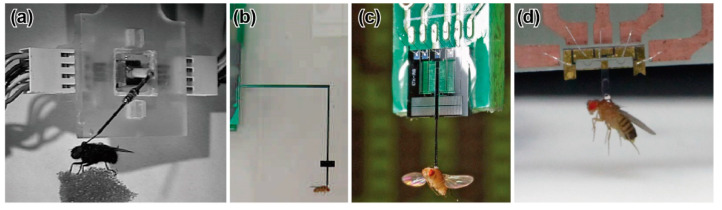
Photographs of flies attached to force probes. (**a**) Blowfly on a biaxial force probe. Reprinted/adapted with permission from [112]. 2022, IEEE. (**b**) Fruit fly on a biaxial MEMS piezoresistive force probe. Reprinted/adapted with permission from [30]. 2022, IEEE. (**c**) Fruit fly on a uniaxial MEMS capacitive force probe. Reprinted/adapted with permission from [33]. 2022, IEEE. (**d**) Fruit fly on a triaxial MEMS piezoresistive force probe. Reprinted/adapted with permission from [37]. 2022, IEEE.

R. J. Wood and R. S. Fearing developed a force probe for measuring the flight force of a micromechanical flying robot that was inspired by an insect [112]. The developed force probe was composed of a cantilever-based strain sensor. The sensor concept is to measure the forces generated on the flapping wing by placing sensors on the wing spars; thus, the force is measured directly. By attaching semiconductor strain gauges to the spar in two directions, the biaxial force can be measured. In addition, the authors proposed a similar type of force probe to measure the total flight force applied to the body. The force probe was composed of two dual cantilever-type sensing elements that could measure the deformation of the cantilever via strain gauges. The resistance changes of the strain gauges were measured via a bridge and amplifier circuit, similar to general resistance sensors. By convolving two sensor elements orthogonally, the biaxial force could be measured. The force resolution and resonant frequency of the developed force probe were 40 μN and 325 Hz, respectively (Table 2). As a demonstration, a blowfly (*Calliphora*) was tethered to the developed force probe using methods similar to those of the M. H. Dickinson group’s research for *Drosophila*, as shown in Figure 6a. The experimental results showed that the measured force varied according to the flapping motion, at a flapping frequency of 160 Hz. Additionally, the blowfly produced a maximal force of 15 mN, which was as much as 12 times its body weight. The force probe was custom, handmade, and structurally similar to the force plates that measure GAF in Section 3.3. In the literature, the flight force measurement was just a demonstration, and a blowfly was selected as a suitable size for the measurement. Thus, the blowfly size is considered the limitation of the size and measurable force range of the handmade force probe.

### 4.3. Early MEMS Piezoresistive Force Probes

One of the first MEMS force probes for insects was proposed by the M. H. Dickinson group [30]. The target insect here was also a fruit fly. The proposed force probe was designed to be L-shaped, and four piezoresistors were formed on the L-shaped beam surface to measure the lift, thrust, and yaw forces, as shown in Figure 5c. The widths of the beams close to and far from the tip were 250 μm and 400 μm, respectively. The force probe was fabricated on a Si wafer. As the piezoresistive layer, an N-doped poly-Si layer was deposited on the wafer, and the fabrication process was similar to that of the MEMS piezoresistive force plates described in Section 3. The fabricated force probe chip was mounted on a substrate and wire bonded. Then, the piezoresistors were connected to a bridge and amplifier circuit. The authors mentioned that forces of 100 μN were measured by attaching small weights at the tip of the sensor, and the resonant frequencies were higher than the flapping frequency, typically approximately 200 Hz (Table 2). As the initial experiment, a fruit fly (*Drosophila melanogaster*) was tethered to the fabricated force probe, as shown in Figure 6b. Then, the force measurement was conducted while the fly was flapping. However, the authors concluded that, even though the sensor signal was observable, the actual flight forces in a single flapping cycle were too small to be measured with the force probe. There was a possibility that the noise would become sufficiently low by averaging the flapping cycles, similar to the approaches of M. H. Dickinson and K. G. Götz [109] mentioned in Section 4.2. The authors also suggested that the sensor should have a force resolution of at least 0.1 μN, in the range of less than 50 μN, in order to measure the flight forces produced by a fruit fly. One of the reasons for the low sensitivity is that the piezoresistive layer is formed on the surface Si layer, while the in-plane directional deformation, i.e., *F*_lift_ and *F*_thrust_, is the measurement target. This problem is similar to the discussion in Section 3.4, which is about the early MEMS force plate for a cockroach. With a single surface piezoresistive layer, it is difficult to measure the multi-axis flight force of “mg”-mass insects.

### 4.4. MEMS Capacitive Force Probes

The B. J. Nelson group developed MEMS highly sensitive capacitive force probes for a fruit fly [31,32,33,34,35]. They also measured the cellular force using the same force probe. The force probe was composed of a Si cantilever and supporting spring beams, so that the cantilever deformed in the in-plane longitudinal direction, as shown in Figure 5d. A comb structure was formed behind the cantilever. A comb structure was also formed in the surrounding area, so that capacitive elements were realized. Provided that the cantilever deforms due to a longitudinal force, the gap between the two comb structures changes. By detecting the capacitive change of the comb structures, the applied force can be measured. The width, length, and thickness of the probe were designed to be 50 μm, 50 μm, and 3 mm, respectively. The width of the comb structures was 5 μm. The force probe was fabricated on an SOI wafer of a device Si layer of 50 μm. The sensor structure was fabricated by a common DRIE etching process. The capacitances of the sensor were connected to a buffer amp and synchronous demodulator circuit. The force resolution and measurable range were 0.68 μN and ±1 mN, respectively (Table 2). The authors also mentioned that the bandwidth, which corresponded to the resonant frequency, was 7.8 kHz. A fruit fly (*Drosophila melanogaster*) was attached to the tip of the fabricated force probe, as shown in Figure 5c. Thus, the measured force corresponded to the lift directional flight force. The experimental results demonstrated that the measured flight force was periodic at a fundamental frequency of approximately 200 Hz, which corresponded to the flapping frequency of fruit flies. In addition, the averaged force in one cycle was 9.3 μN, which corresponded to the range of the typical body weights of fruit flies.

The MEMS capacitive force probe, described above, was a single-axis sensor. The B. J. Nelson group also developed MEMS multi-axis force probes utilizing sophisticated comb structures [113,114]. By forming the units of the comb structures in the orthogonal directions, the in-plane two-axis force could be detected. In addition, by utilizing a double SOI wafer with two overlapping device Si layers, a vertical directional gap could be applied to the comb structures, so that the out-of-plane axis force became detectable. The authors reported that the force resolution was less than 0.2 μN, with a measurable range of approximately ±200 μN. Using multi-axis force probes, the flight forces of a fruit fly during flapping motion in three-dimensions can, in principle, be measured.

Capacitor-type sensors realize high force sensitivity. Therefore, the high sensitivity and wide measurement range can be simultaneously satisfied. Meanwhile, the DC component is difficult to measure, due to the detection principle. Therefore, even if the variation of the force during one flapping cycle can be revealed, it is difficult to evaluate its absolute value.

In the case of a capacitive element, the dimensional sensitivity characteristics are contrary to those of piezoresistive element. In-plane deformations can be measured with high sensitivity, but out-of-plane deformations are not suitable for detection because the capacitive element of a simple comb electrode does not distinguish positive or negative sensing in the out-of-plane direction. Therefore, to realize a multi-axis force probe with more than three axes, one must conduct complex processes on an expensive double SOI wafer.

### 4.5. MEMS Piezoresistive Multi-axis Force Probes

The H. Takahashi and I. Shimoyama group developed MEMS force probes for a fruit fly [36,37], in addition to the force plates described in Section 3. The developed force probe was based on a piezoresistive-type multi-axis force probe previously reported by their group [115], the fundamental principle of which was the same as that for their MEMS force plates. The force probe was composed of a Si cantilever and four supporting beams, as shown in Figure 5e. Two beams had sidewall-doped piezoresistors to detect the *x-y*-axis in-plane force applied to the tip of the cantilever, while one beam has a surface-doped piezoresistor to detect the *z*-axis out-of-plane force. The other beam worked as the electric ground. Thus, the triaxial force applied to the tip of the cantilever could be detected by measuring the resistance changes of the three beams at the same time. Since the responses to the triaxial force were orthogonal to each other, low crosstalk could be realized.

The width, length, and thickness of the probe were designed to be 360 μm, 1.4 mm, and 50 μm, respectively. The force probe was fabricated on an SOI wafer of a device Si layer of 50 μm. The fabrication process of the force probe was almost the same as that of the MEMS force plate array for ants [25]; only the mask pattern was different. The piezoresistors of the sensor were connected to a bridge and amplifier circuit with a common electric ground. The force resolutions along the *x-*, *y-*, and *z*-axes were 0.2 μN, 0.3 μN, and 0.6 μN, respectively, in the range of ±120 μN (unpublished data) (Table 2). A fruit fly (*Drosophila melanogaster*) was attached to the tip of the fabricated force probe, as shown in Figure 6d. Then, the resonant frequency of the force probe with a fruit fly was measured to be 680 Hz along the *z*-axis, which was the lowest stiffness axis (unpublished data). The experimental results showed the triaxial flight force during the flapping motion. The measured flight force was demonstrated to be synchronized with the flapping motion of the fruit fly.

As mentioned in Section 3.6, the fabrication process of the force probe, including the sidewall doping method, is currently not a common MEMS process. Unlike the ant’s force plate array, the force probe is supported from both sides by the thin beams, which is not a free-end cantilever structure. Thus, the sensor structure is more fragile, and there is constantly the possibility of breakage during experiments, such as fly attachments. Therefore, it may be difficult to realize a higher sensitivity with the sensor design.

### 4.6. Differential Pressure Sensors for Insects

**Figure 7:**
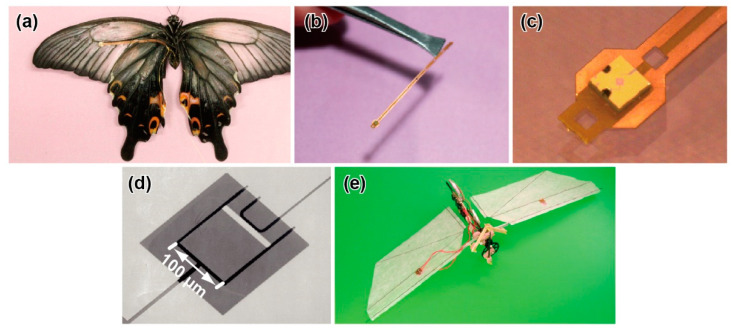
Photograph of a butterfly with a MEMS differential pressure sensor. (**a**) Butterfly with the sensor attached to the ventral surface of the wing. Reprinted/adapted with permission from [117]. 2022, IOP publishing. (**b**,**c**) Cu/polyimide flexible electrode with the sensor chip. Reprinted/adapted with permission from [117]. 2022, IOP publishing. (**d**) SEM image of the piezoresistive cantilever. Reprinted/adapted with permission from [116]. 2022, IOP publishing. (**e**) Photograph of an insect-like ornithopter with the sensor. Reprinted/adapted with permission from [118]. 2022, IOP publishing.

The H. Takahashi and I. Shimoyama group developed not only MEMS force probes, but also MEMS differential pressure sensors for butterflies [116,117]. The MEMS differential pressure sensor was composed of a piezoresistive cantilever 125 μm × 100 μm × 0.3 μm in size (Figure 7d). The fundamental principle was similar to that of their MEMS force plates and force probe described above. Thus, the fabrication process was also similar. The piezoresistive layer was formed on the surface of the cantilever. The cantilever deformed when differential pressure was applied between the upper and lower surfaces of the cantilever. Due to the micron-size gap surrounding the cantilever, air leakage from the gap was sufficiently low, such that the cantilever deformed as theoretically expected, without being affected by the leakage. The sensor realized a pressure resolution less than 0.1 Pa, due to its highly sensitive piezoresistance and sub-micron thick cantilever structure in the range of −20~+20 Pa (Table 3). The resonant frequency of the cantilever was over 10 kHz, which was sufficiently higher than the flapping frequency. Additionally, the sensor did not respond to acceleration, due to the sub-micron thickness. Thus, even if the sensor is attached to the wing surface, there is little influence of the acceleration from the flapping motion.

The aerodynamic force was measured by attaching the sensor chip to the wing surface, at the point where a through hole penetrated, as shown in Figure 7a–c. As the target insect, a spangle butterfly (*Papilio protenor*) was used because of both the large wing area and heavy weight among lepidopterans; it can tolerate the additional weight of the sensor system. A flexible electrode was used for the sensor attachment. A sensor chip of 1 mm × 1 mm × 0.3 mm in size was attached to the edge of the electrode, while two Au wires were connected to the opposite side of the electrode. Then, the electrode was adhered to the vein of the wing. In terms of weight, the sensor chip was 0.7 mg, which was small enough for a single wing. The total weight, including the Au wires, was less than 10% of the body weight (Table 3), which was thought to be within the acceptable range, considering that the food is approximately 1/10 of the body weight. Using the sensor-attached butterfly, the differential pressure on the wing was measured during the take-off motion. At the point of the centre of the forewing, periodic and symmetric differential pressure was induced, according to the upstroke and downstroke. The maximum differential pressure reached approximately 10 Pa, which was 10 times larger than the wing load.

Aerodynamic force measurement, using MEMS differential pressure sensors, has been adapted to insect-like ornithopters, as well as to an actual butterfly. The advantages of using insect-modelled ornithopters are that we can focus on and extract the characteristic points in flight performance and that reproducible experiments can be easily conducted. Figure 7e shows an ornithopter modelled on a hawk moth, and the MEMS differential pressure sensor was attached to the wing surface [118]. Because of its large payload, a datalogger, including an amplifier circuit and a battery, was mounted on the ornithopter to record the sensor data. Thus, the measurement could be conducted in completely free flight. The sensor system was applied, not only to the moth-modelled ornithopter, but also to a dragonfly-modelled ornithopter, with different phase lags between the forewings and hindwings [119], as well as a beetle-modelled ornithopter with fixed forewings [120]. In each study, the differential pressure during flight was measured, and the aerodynamic performances were evaluated when the characteristic parameters were varied.

Additionally, by placing the MEMS differential pressure sensors on the ground, the ground pressure caused by the downstroke when an insect took off was detected [121]. In the experiment, a spangle butterfly (*Papilio protenor*) was also used. The maximum pressure was approximately 1 Pa. The local maximum pressure was generated on both the downstroke and upstroke. The measurement system could allow us to evaluate the ground effect with tiny differential pressure [122].

In these studies, only spangle butterflies were tested as actual insects. At the present stage, it is difficult to apply this method to insects smaller than spangle butterflies. The bottleneck is not the MEMS differential pressure sensor chip, but the wiring. The wiring becomes a non-negligible size to the wing and inhibits the flapping motion. The authors attached the same setup to a swallowtail butterfly, which is approximately 200 mg; then, the butterfly could not take off (unpublished). If thinner wiring is available, the sensor system is applicable to smaller insects. The MEMS cantilever structure, which is the sensing element, is on the order of 100 μm; thus, the size can be further miniaturized, in principle. However, the manual post-process is the size limitation.

### 4.7. Wearable Sensor for Wing

Although they do not directly measure the force, wing wearable sensors have been developed to measure the flapping frequency [123]. The sensor is composed of a thin silicone polymer film with silver particles, so that the resistance changes when a bending deformation occurs. The film was 0.25 mm thick. The fabricated sensor was attached to the wings of a tethered silk moth and a tethered dragonfly. Then, the flapping frequency was obtained by measuring the change in resistance when the wings flapped. While it is less computationally expensive than high-speed camera images, it is more invasive because the sensor is directly fixed to the wing. Especially, it is currently necessary to attach electrodes via wires. If the target insect is tethered, the force probe is considered more suitable for measuring the flapping frequency because the calculation process cost is similar. However, if it is possible to be completely wireless and measure more localized strain, the wearable sensor will be useful for measuring the distribution of the wing deformation during the flapping motion.

## 5. Challenges and Future Work

We can summarize that, at present, one of the powerful methods for measuring the tiny forces produced by tiny insects is to develop a specialized sensor device utilizing MEMS technology. This is because the MEMS force sensor devices are precisely processed on the order of micrometres, including array structure and highly sensitive sensing elements, such as piezoresistors, which can be formed at any desired location and are difficult to develop by other fabrication technologies. However, there are restrictions on the MEMS fabrication process. For example, provided that the sensor structure is fabricated on an SOI wafer, freely making changes in the thickness direction from location to location is difficult; the thickness of the device Si layer is normally on the order of 0.1 μm to 100 μm. A complete 3D structure, such as a spring coil, cannot be fabricated. The introduced MEMS sensors have been designed and fabricated under these restrictions. As the sensing elements of MEMS force sensors, the wireless sensing element [124] or highly sensitive piezoelectric element [125] has been developed, as well as conventional methods. These MEMS force sensors have not been used for force measurements in insects (or just demonstration), but they are expected to be the platform. In addition to the MEMS sensor sheets introduced in Section 3.9.2, flexible and stretchable MEMS sensors with non-Si sensing elements are under development. For example, micro-3D-printed structures with conductive graphene ink [126] and laser-induced graphene films [127,128] are attracting attention as next-gen sensing element materials. These sensing elements are soft, highly sensitive, and break-proof. Therefore, force sensor for tiny insects, which have not been measured, are expected to be developed.

In this paper, we mainly introduced force plates, force probes, and differential pressure sensors as specialized sensors to measure the tiny forces exerted by the locomotion of airborne and terrestrial insects, with a focus on the methodology. However, the introduced methods are not all-inclusive of insects. For example, many species in water use hydrodynamic force for locomotion. The hydrodynamic force of aquatic animals is similar to the aerodynamic force of flying animals. The hydrodynamic force acts at the boundary surface between driving parts and water. An aquatic insect, such as a diving beetle, swims by paddling its legs, similar to flying insects flapping their wings [129]. Additionally, some small insect larvae twist their body to swim, similar fish fins [130]. Previously, there have been a few reports on micro-force sensors to measure the swimming hydrodynamic force of small insects in water. However, several MEMS force sensors have been developed for swimming animals. In biologging research, pressure and flow sensors utilizing MEMS technology have been developed to measure the swimming depth and waterflow velocity by attaching several sensor units to the bodies of marine animals [131,132]. Meanwhile, swimming animals have sensory organs on their body surfaces that detect hydrodynamic forces, which inspired the development of several MEMS force sensors [133,134]. Such sensors are applied to bioinspired robots [135]. The most significant reason for the difficulty in measuring the hydrodynamic forces of small insects in water is the underwater measurement environment. The sensor must be electrically waterproof, and waterproof covering material will make the sensor and wiring larger. Additionally, it is difficult to glue the sensors to insects in water because of the limitations of adhesives. While it is possible to apply the process to large fish, it is difficult for small insects. However, cell measurements have been performed in water, so these technical issues will be solved in the future.

Additionally, although we focused on a method to measure the tiny forces produced by tiny insects, the measurement method can also be applied to robots inspired by such tiny insects, e.g., the ornithopter with a MEMS differential pressure sensor, introduced in Section 4.6. For example, a micro-force plate can measure the GRF of a micro-multilegged robot during walking [136]. Moreover, it is known that insects, themselves, have sensors, such as campaniform sensilla and stretch receptors [137,138] for motion control, which is similar to the swimming animals introduced above. Therefore, it is also expected that MEMS-based micro-force sensors can be directly applied to robots for more sophisticated control.

Currently, not many researchers are involved in this field because the expertise of both sensor fabrication processes and biomechanics are required. When the barriers to accessing advanced technologies, including MEMS, decreases, the research focus will shift from developing devices to a comprehensive understanding of the tiny forces acting on insects, and many researchers will use the micro-force sensor devices as a platform.

## Figures and Tables

**Table 2:** Specifications of the developed force probes. The force probes were designed according to the size and motion of the measurement target.

	Target Animal/Insect	Probe Length	Sensing Element	Force Direction	Force Resolution	Resonant Frequency
M. H. Dickinson and K. G. Götz (1996) [109]	Fruit fly 1 mg	60 mm	Photodiode	*x*, *z*	No data	4.0 kHz
M. H. Dickinson and J. R. B. Lighton (1995), F. O. Lehmann and M. H. Dickinson (1997) [110,111]	Fruit fly 1 mg	10 mm	Photodiode	1 axis	No data	No data
R. J. Wood and R. S. Fearing (2001) [112]	Blowfly 100 mg	5 mm	Semiconductor Strain gauge	*x*, *z*	40 μN	325 Hz
M. Nasir et al. (2005) [30]	Fruit fly 1 mg	No data	Piezoresistive element	*x*, *z*, *M*_z_	<100 μN	>200 Hz
Y. Sun et al. (2005, 2007), C. Graetzel et al. (2008, 2008, 2010) [31,32,33,34,35]	Fruit fly 1 mg	3 mm	Capacitive element	*z*	0.68 μN	7.8 kHz
K. Azuma et al. (2012, 2013) [36,37]	Fruit fly 1 mg	1.4 mm	Piezoresistive element	*x*, *y*, *z*	<0.6 μN (unpublished)	680 Hz (unpublished)

**Table 3:** Characteristics of the butterfly specimen and differential pressure sensor.

Spangle Butterfly		Differential Pressure Sensor	
Wing length	57 mm	Sensor size	1 mm × 1 mm × 0.3 mm
Wing area	3.6 mm^2^	Total weight	35 mg
Total weight	433 mg	Sensor chip weight	0.7 mg
Single wing weight	50 mg	Resonant frequency	13 kHz
Flapping frequency	8.5 Hz	Measurement range	−20~+20 Pa
Wing load	1.2 Pa	Resolution	0.1 Pa

## Data Availability

No new data were created or analyzed in this study. Data sharing is not applicable to this article.
